# Roles of Mitochondrial Sirtuins in Mitochondrial Function, Redox Homeostasis, Insulin Resistance and Type 2 Diabetes

**DOI:** 10.3390/ijms21155266

**Published:** 2020-07-24

**Authors:** Chih-Hao Wang, Yau-Huei Wei

**Affiliations:** 1 Section on Integrative Physiology and Metabolism, Joslin Diabetes Center, Harvard Medical School, Boston, MA 02215, USA; chih-hao.wang@joslin.harvard.edu; 2 Center for Mitochondrial Medicine and Free Radical Research, Changhua Christian Hospital, Changhua City 50046, Taiwan; 3 Institute of Clinical Medicine, National Yang-Ming University, Taipei 11221, Taiwan

**Keywords:** sirtuins, mitochondrial dysfunction, insulin resistance, diabetes, oxidative stress, metabolism, adipogenesis, antioxidant defense

## Abstract

Mitochondria are the metabolic hubs that process a number of reactions including tricarboxylic acid cycle, β-oxidation of fatty acids and part of the urea cycle and pyrimidine nucleotide biosynthesis. Mitochondrial dysfunction impairs redox homeostasis and metabolic adaptation, leading to aging and metabolic disorders like insulin resistance and type 2 diabetes. SIRT3, SIRT4 and SIRT5 belong to the sirtuin family proteins and are located at mitochondria and also known as mitochondrial sirtuins. They catalyze NAD^+^-dependent deacylation (deacetylation, demalonylation and desuccinylation) and ADP-ribosylation and modulate the function of mitochondrial targets to regulate the metabolic status in mammalian cells. Emerging evidence has revealed that mitochondrial sirtuins coordinate the regulation of gene expression and activities of a wide spectrum of enzymes to orchestrate oxidative metabolism and stress responses. Mitochondrial sirtuins act in synergistic or antagonistic manners to promote respiratory function, antioxidant defense, insulin response and adipogenesis to protect individuals from aging and aging-related metabolic abnormalities. In this review, we focus on the molecular mechanisms by which mitochondrial sirtuins regulate oxidative metabolism and antioxidant defense and discuss the roles of their deficiency in the impairment of mitochondrial function and pathogenesis of insulin resistance and type 2 diabetes.

## 1. Introduction

Diabetes present high level of glucose in the blood circulation and systemic metabolic abnormalities. Diabetes is divided into type 1 and type 2, which are characterized by insulin deficiency or the defective insulin response [1]. Insulin is secreted by pancreatic β cells upon glucose stimulation, and insulin can subsequently lower blood glucose and fatty acids levels by inhibition of gluconeogenesis in the liver, repression of lipolysis in adipose tissues and stimulation of glucose uptake in adipose tissues and muscle cells. Type 1 diabetes, also known as juvenile-onset diabetes, results from the loss of insulin-secreting pancreatic β cells via an autoimmune attack. Type 2 diabetes is caused by insensitivity or resistance of insulin action in the peripheral tissues [1]. Most importantly, chronic hyperglycemia induces inflammation and oxidative stress, causing damages and dysfunction in several tissues and resulting in diseases including peripheral neuropathy, cardiovascular disease, retinopathy, nephropathy and sexual abnormalities. Recent increase in the incidence of type 2 diabetes worldwide is due to an unhealthy lifestyle, reduced exercise and aging in modern society.

Mitochondria generate cellular energy in response to different nutrient stimulation and produce reactive oxygen species (ROS) simultaneously to trigger a stress response under normal circumstances. One of the mitochondrial functions is to host a couple of metabolic reactions including tricarboxylic acid cycle (TCA) cycle, fatty acid oxidation, ketogenesis and part of the urea cycle and heme biosynthesis [2]. Thus, mitochondria contain many α-keto acids, lipid intermediates and intermediary metabolites such as acetyl-CoA, acryl-CoA, NAD^+^/NADH, NADP^+^/NADPH and ADP/ATP, which can modulate the activities of multiple enzymes [3]. Given the unique metabolic roles of mitochondria, dysfunction of the organelles impairs cellular metabolism that contributes to aging, neurodegenerative diseases, metabolic disorders and cancer. Accumulated evidence suggests that mitochondrial dysfunction is related to the development of insulin resistance and type 2 diabetes [4,5].

Genetic or pharmacological manipulation of mouse models has clearly shown that abnormalities of glucose and lipid metabolism triggered by mitochondrial defects are the causative factors in the pathogenesis of type 2 diabetes [6,7,8]. Additionally, chronic over-nutrition induces imbalance of cellular metabolism and pressures mitochondrial bioenergetic process, leading to elevation of ROS production. Oxidative stress derived from mitochondrial overload is well known as an underlying mechanism of insulin resistance [9]. ROS accumulation resulted from impairment of respiratory enzymes and antioxidant defense system dysregulates the insulin-stimulated signaling cascade, glucose and fatty acid utilization, insulin secretion as well as inflammation in a myriad of cell types. Insulin receptor substrate 1/2 (IRS1/2) is phosphorylated by the tyrosine kinase of the insulin receptor in response to insulin binding. Oxidative stress induces the activation of several serine kinases, such as JNK, PKC and IKKβ, and increases serine phosphorylation of IRS1/2. Serine phosphorylation of IRS1/2 antagonizes the action of tyrosine phosphorylation and in turn reduces the activation of phosphatidylinositol 3-kinase (PI3K) leading to abrogate the insulin signaling [9]. Taken together, mitochondrial dysfunction is a hallmark of insulin resistance and type 2 diabetes [10]. Targeting to enhancement of mitochondrial function and promotion of antioxidant capacity may lead to the development of novel therapeutic strategies to treat insulin resistance and type 2 diabetes.

**Figure 1:**
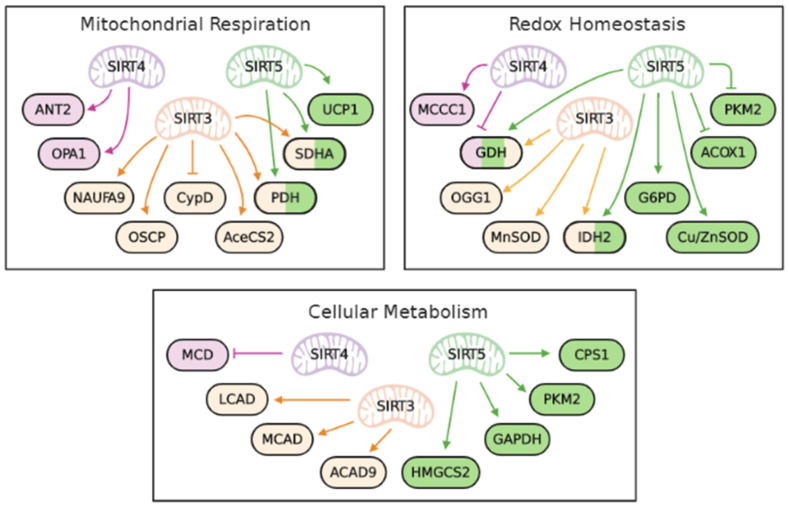
Mitochondrial sirtuins regulate mitochondrial respiration, redox homeostasis and cellular metabolism. Mitochondrial sirtuins, SIRT3, SIRT4 and SIRT5, coordinately regulate the function of proteins involved in mitochondrial respiration, redox homeostasis and cellular metabolism. They activate or inhibit the functions of target proteins or enzymes via deacetylation (SIRT3-5), ADP-ribosylation (SIRT4), desuccinylation (SIRT5) and malonylation (SIRT5). They not only modulate the same proteins by different post-translational modifications but also target multiple proteins to orchestrate the regulation of metabolic pathways as a whole. Abbreviations: ACAD9, acyl-CoA dehydrogenase 9; AceCS2, acetyl-CoA synthetase; ACOX1, acyl-CoA oxidase 1; ANT2, adenine nucleotide translocator 2; CPS1, carbamoyl phosphate synthetase 1; Cu/ZnSOD, Cu/Zn-containing superoxide dismutase; CypD, cyclophilin D; G6PD, glucose 6-phosphate dehydrogenase; GAPDH, glyceraldehyde 3-phosphate dehydrogenase; GDH, glutamate dehydrogenase; HMGCS2, 3-hydroxy-3-methylglutaryl-CoA synthase 2; IDH2, isocitrate dehydrogenase 2; LCAD, long-chain acyl-CoA dehydrogenase; MCAD, medium-chain acyl-CoA dehydrogenase; MCCC1, methylcrotonyl-CoA carboxylase 1; MCD, malonyl-CoA decarboxylase; MnSOD, manganese-containing superoxide dismutase; NDUFA9, NADH:ubiquinone oxidoreductase subunit A9; OGG1, 8-oxoguanine-DNA glycosylase 1; OPA1, optic atrophy 1; OSCP, oligomycin-sensitivity conferring protein; PDH, pyruvate dehydrogenase; PKM2, pyruvate kinase muscle isozyme 2; SDHA, succinate dehydrogenase flavoprotein subunit A; UCP1, uncoupling protein 1.

In mammalian cells, there are 7 sirtuins, SIRT1-7, which are involved in the regulation of multiple cellular metabolic pathways by post-translational modification [11]. Sirtuins are evolutionarily conserved from yeasts to humans and regulate metabolism by histone deacetylation and chromatin silencing. Sirtuins display different cellular localization and multiple enzymatic functions in the presence of nicotinamide adenine dinucleotide (NAD^+^), which include ADP-ribosylation, deacetylation, demalonylation, desuccinylation, deglutarylation, demyristoylation and depalmitoylation. SIRT1 and SIRT2 are deacetylases mainly localized to the nucleus and cytoplasm, respectively. SIRT6 and SIRT7 are nuclear ADP-ribosylase and deacetylase, respectively. Importantly, SIRT3–5 are primarily localized to the mitochondrial matrix and regulate the function of many mitochondrial proteins [12]. SIRT1 has been proposed to play an important role in insulin resistance by transcriptional regulation of specific genes [13]. Considering the crucial role of mitochondria in energy metabolism, redox homeostasis and processing lots of biochemical reactions, it is logical to conjecture that mitochondrial sirtuins play vital roles in aging and metabolic adaptation. Emerging evidence indicates that defects in mitochondrial sirtuins contribute to abnormalities of glucose and lipid metabolism in mice and human subjects with insulin resistance [12,14,15,16]. Therefore, in this review, we focus on the mechanisms underlying SIRT3, SIRT4 and SIRT5-mediated regulation of mitochondrial function and metabolism (Figure 1), and also discuss the implication of their deficiency in the pathogenesis of insulin resistance and type 2 diabetes.

## 2. SIRT3 Regulates Mitochondrial Metabolism and Insulin Sensitivity

SIRT3 is a NAD^+^-dependent deacetylase localized to mitochondria. Several studies clearly demonstrated the roles of SIRT3 in the regulation of mitochondrial respiratory function, redox homeostasis, metabolic adaptation, insulin response and stem cell differentiation [17]. Polymorphisms, expression level and enzyme activity of SIRT3 have been highly correlated with longevity and metabolic syndrome in the human. SIRT3 deficiency has been implicated in the pathogenesis of aging and age-related metabolic dysfunction such as cardiovascular disease, fatty liver, insulin resistance and type 2 diabetes [15,17,18].

### 2.1. SIRT3 in Mitochondrial Function

SIRT3 promotes mitochondrial function by deacetylation and activation of respiratory enzymes. Loss of SIRT3 reduces intracellular ATP level in mouse embryonic fibroblasts, heart, kidney and liver of mice [19]. SIRT3 can directly deacetylate a subunit of Complex I, NDUFA9 via direct protein interaction. Mitochondria from SIRT3-deficient mice revealed profound and selective defects in respiratory enzymes. Addition of exogenous SIRT3 could enhance mitochondrial respiration by increasing Complex I activity [19]. In addition, Cimen et al. identified the flavoprotein subunit A of succinate dehydrogenase (SDHA) in Complex II as a substrate of SIRT3 [20]. Comparison of acetylation profiles in mitochondrial proteins from wild-type and SIRT3 KO mice led to the identification of changes in acetylation on several lysine residues in SDHA. Based on the crystal structure of SDHA, acetylation of those lysine residues would block the substrate accessibility of succinate dehydrogenase (SDH). However, deacetylation by SIRT3 opens the pocket of the active site and promotes enzymatic activity of SDH [20]. On the other hand, our group showed that SIRT3 interacts with oligomycin-sensitivity conferring protein (OSCP), a subunit of Complex V (ATP synthase), and enhances its activity [21]. The expression of SIRT3 is reduced in the primary culture of skin fibroblasts of patients with large-scale mtDNA deletion or chronic progressive external ophthalmoplegia (CPEO) syndrome. Oxidative stress from these pathogenic mtDNA mutations decreases SIRT3 expression, leading to a decline of respiratory enzyme function via post-translational modification [21].

Several studies have revealed that SIRT3 modulates the generation of intermediary metabolites to be used as a source of energy. SIRT3 can deacetylate and activate pyruvate dehydrogenase (PDH) to facilitate the conversion of pyruvate to acetyl-CoA [22]. That shifts glucose utilization from anaerobic glycolysis to aerobic metabolism. Additionally, SIRT3 increases the pool of acetyl-CoA from acetate by activating acetyl-CoA synthetase (AceCS2) in the prolonged starvation status [23,24]. Taken together, SIRT3 plays a role in the regulation of metabolic flexibility and coordinates the switch between different metabolic pathways in response to energy demand.

SIRT3 modulates mitochondrial permeability transition pore (mPTP) via deacetylation of cyclophilin D (CypD), which is a regulatory protein of mPTP. Cardiomyocytes with SIRT3 deficiency is prone to open the mPTP and trigger apoptosis in response to oxidative stress [25]. It was found that mice with SIRT3 KO display accelerated age-dependent mitochondrial swelling and aortic constriction-induced heart failure or fibrosis [25]. Interestingly, the deacetylation site of CypD by SIRT3 is near the binding site of cyclosporine A, an inhibitor of CypD. Treatment with cyclosporine A could rescue deleterious phenotypes in SIR3-deficient cardiomyocytes [25]. In addition, SIRT3 is involved in the regulation of mitophagy in response to mitochondrial damage. Overexpression of SIRT3 recovers mitochondrial dysfunction and cardiomyocyte injury in a mouse model of diabetic cardiomyopathy [25]. However, SIRT3-mediated beneficial effects would be attenuated by the inhibition of mitophagy. The underlying mechanism is that deacetylation and activation of FoxO3A by SIRT3 upregulates *Parkin* expression to facilitate mitochondrial quality control by mitophagy [26].

Mitochondria are able to modulate influx and efflux of Ca^2+^ ions to alter both the amplitude and the spatiotemporal distribution pattern of the intracellular Ca^2+^ levels. Mitochondrial Ca^2+^ uniporter (MCU) machinery facilitates the entry of Ca^2+^ ions to the matrix. H^+^/Ca^2+^ and Na^+^/Ca^2+^ exchangers (NCX) efflux Ca^2+^ ions from the matrix to the cytosol. Tight regulation of these proteins is important to increase the Ca^2+^ level to activate mitochondrial enzymes and to prevent accumulation of Ca^2+^ ions and Ca^2+^ overload within the mitochondria [27]. It has been proven that dysregulation of Ca^2+^ homeostasis is related to metabolic diseases such as obesity, insulin resistance and type 2 diabetes in the human and animals [28]. Higher intracellular Ca^2+^ level has been found in primary adipocytes isolated from obese human subjects with insulin resistance and diabetic rats [29,30]. It has been demonstrated that SIRT3 protects cortical neurons from oxidative stress-induced mitochondrial Ca^2+^ overload. Knockdown of SIRT3 exacerbates H_2_O_2_-induced cell death while overexpression of SIRT3 attenuates Ca^2+^ overload in mitochondria after H_2_O_2_ treatment [31]. In addition, SIRT3 inhibits the expression of *MCU* by reducing the level of H3K27ac on the promoter region via the AMPK-dependent pathway. Downregulation of MCU by SIRT3 overexpression leads to the alleviation of the excess Ca^2+^ uptake into mitochondria and detrimental effects in brown adipocytes of mice treated with the high-fat diet [32]. Moreover, as above-mentioned, the role of SIRT3 in regulating mPTP opening may contribute to the modulation of mitochondrial Ca^2+^ homeostasis.

### 2.2. SIRT3 in Redox Homeostasis

Imbalance of redox homeostasis and accumulation of excess ROS are major causes of aging and metabolic disorders such as insulin resistance and diabetes. Emerging evidence has substantiated the notion that there is a link between SIRT3 and redox homeostasis regulation [17]. It has been proved that SIRT3 is required for the beneficial effects of caloric restriction to reduce the intracellular ROS levels in aging, obesity and diabetes [15,33].

Sirt3 has been shown to directly interact with 8-oxoguanine-DNA glycosylase 1 (OGG1), the enzyme responsible for the repair of the 8-hydroxy-2′-deoxyguanosine (8-OHdG) in DNA. SIRT3 deacetylates OGG1 and protects it from degradation and leads to promoting the repair of oxidative DNA damage, especially in mitochondrial DNA (mtDNA). Silencing SIRT3 causes more severe damage to mtDNA and nuclear DNA and triggers the cells to undergo apoptosis in response to oxidative stress challenge [34]. In the diabetic Zuker obese rat model, a decrease in SIRT3 activity and mitochondrial function was accompanied with a high level of 8-OHdG in the blood circulation and urine compared to non-diabetic lean rats [35]. Proximal tubular cells cultured in high glucose medium revealed defective mitochondrial morphology, inactivation of SIRT3 and reduction of the antioxidant capacity. These results indicate the importance of SIRT3 in redox homeostasis, especially to combat the oxidative stress induced by the high glucose/lipid environment of diabetic tissues [35].

Mice with SIRT3 deficiency displayed increased acetylation and reduced activity of superoxide dismutase 2 (SOD2), which is also known as MnSOD [36]. MnSOD is a mitochondrial antioxidant enzyme for converting the superoxide anion (O_2_^−^) to hydrogen peroxide (H_2_O_2_), which can be further detoxified to H_2_O by catalase. MnSOD serves as the first line O_2_^−^scavenger to cope with oxidative stress induced by electron leakage from mitochondrial respiratory chain. Two important lysine residues, K53 and K89, of MnSOD have been identified as direct target sites of SIRT3. Mutations of these two residues block deacetylation of MnSOD and lead to sensitizing ROS accumulation and mitochondrial dysfunction in the cells exposed to oxidative stress [37]. Moreover, Tao et al. also demonstrated that SIRT3 could deacetylate another two lysine residues, K68 and K122, on MnSOD and regulate its antioxidant activity [38]. Notably, in addition to its regulatory function on enzymatic activity, SIRT3 is involved in the induction of *MnSOD* and *Catalase* expression by the activation of FoxO3A transcriptional activity [39]. This has been demonstrated in SIRT3-mediated amelioration of hypertrophy in primary cardiomyocytes [39]. Foxo3A-dependent upregulation of the protein levels of MnSOD and catalase leads to a decrease of the intracellular ROS levels and suppression of Ras/MAPK/ERK activation.

In addition to MnSOD, SIRT3 also targets and activates mitochondrial isocitrate dehydrogenase 2 (IDH2) via deacetylation [40]. IDH2 promotes antioxidant capacity in mammalian cells by increasing the NADPH level and the ratio of reduced GSH to oxidized GSSG. Under caloric restriction, it was found that SIRT3-mediated IDH2 activation was required for preventing cell death in aging [40]. The deacetylation of lysine 413 on IDH2 is regulated by SIRT3 to promote its antioxidant function. Genetic manipulation of IDH2 to mimic deacetylated lysine on 413 (K413R) dramatically increases the IDH2 activity and protects against oxidative stress in SIRT3 KO mouse embryonic fibroblasts [41]. In contrast, incorporation of acetyl-lysine to IDH2 at position 413 inactivates the enzyme [41]. Moreover, glutamate dehydrogenase (GDH) is deacetylated and activated by SIRT3. Then GDH promotes the production of NAD(P)H by catalysis of the conversion of glutamate to α-ketoglutarate [42].

### 2.3. SIRT3 in Glucose Uptake, Insulin Sensitivity and Fatty Acid Metabolism

It has been demonstrated that SIRT3 is downregulated in skeletal muscle in both streptozotocin (STZ)-induced diabetic mice and high-fat diet-induced obese mice [43]. These results indicate a role of SIRT3 deficiency in the pathogenesis of insulin resistance and type 2 diabetes. Increasing evidence has shown that SIRT3-deficinet mice displayed insulin resistance and glucose intolerance phenotypes upon feeding with the high-fat diet. Using hyperinsulinemic-euglycemic clamp, a gold standard method, Lantier et al. demonstrated that SIRT3 KO mice displayed a low glucose infusion rate upon exogenous insulin administration, which indicates the insensitivity of glucose uptake by peripheral tissues in response to insulin [44]. Mechanistically, the dramatic decrease of a glucose-dependent oxygen consumption rate is due to mitochondrial dysfunction caused by hyperacetylated respiratory enzymes [44]. Loss of the SIRT3-mediated antioxidant defense function also caused impairment of insulin signaling and glucose uptake in the peripheral tissues due to accumulation of ROS [44].

In addition to glucose metabolism, SIRT3-decificient mice display increased levels of fatty acid intermediates and accumulation of triglycerides resulted from reduced capacity of fatty acid oxidation in liver during fasting. Mass spectrometry analysis clearly identified the enzymes involving fatty acid oxidation as SIRT3 substrates, which include long-chain acyl-CoA dehydrogenase (LCAD), medium-chain acyl-CoA dehydrogenase (MCAD) and acyl-CoA dehydrogenase 9 (ACAD9). These findings suggest that SIRT3 also regulates the lipid utilization and contributes to the elimination of excess fatty acids upon feeding mice with a high-fat diet [45,46].

In addition to the diet-induced obese mouse model, SIRT3 also plays a role in the pathogenesis of streptozotocin (STZ)-induced diabetes. Absence of SIRT3 accelerates the progression of diabetes development and triggers other complications including cardiac problems and interstitial fibrosis [26]. Finley et al. demonstrated that SIRT3-targeted deacetylation and activation of SDH is impaired in the brown adipose tissues (BAT) of SIRT3 KO mice [47]. Considering the high ability in glucose/lipid uptake and metabolism of the BAT, its dysfunction has been strongly correlated with the development of insulin resistance and type 2 diabetes [48]. A recent study has shown the importance of succinate-SDH pathway in promoting energy expenditure of BAT and its anti-obesity effect [49]. This further highlights the regulatory role of SIRT3 in the utilization of excess fuels to generate heat or energy.

### 2.4. SIRT3 in Insulin Secretion

Downregulated expression of SIRT3 has been observed in the isolated islets from patients with type 2 diabetes. High-fat diet results in an increase of ROS and decrease of SIRT3 level in β cells of mice [50]. In addition, IL-1β and TNFα treatments, which mimic the inflammation condition of diabetes, decreased SIRT3 expression in a mouse insulin-producing β cell line (INS1) [51]. INS1 cells with SIRT3 deficiency showed a decrease of β cell identity and functions including downregulation of marker genes and low capacity of insulin secretion [51]. Furthermore, SIRT3 knockdown in the primary culture of islets isolated from SIRT3 KO mice and pancreatic β cell line (MIN6) derived from a mouse insulinoma both revealed a decline of the MnSOD activity and impairment of glucose-stimulated insulin secretion and ATP production [50].

SIRT3 protects β cells from cell death or damage upon oxidative stress challenge. Loss of SIRT3 also renders cells prone to apoptosis and blocks the protective effect of NAD^+^ under inflammation stimulation due to deleterious accumulation of intracellular ROS [51]. Besides, overexpression of SIRT3 mitigates excess lipids-induced ER stress and cell toxicity in β cells [52]. All these findings indicate the potential role of SIRT3 in the function of β cells and its protective effect against high glucose/lipids/ROS environment of insulin resistance and type 2 diabetes.

### 2.5. SIRT3 in the Differentiation of Myocytes and Adipocytes

It has been documented that SIRT3 plays an important role in the maintenance and differentiation of stem cells [53]. Downregulation of SIRT3 inhibits the differentiation ability of aged stem cells with high oxidative stress. Replicative senescence decreases the SIRT3 level and impairs the capacity of adipogenesis and osteogenesis in human bone marrow-derived stem cells. Moreover, restoration of the SIRT3 level not only rescues the senescence phenotype and reduces the intracellular levels of ROS but also improves the differentiation potential of senescent stem cells [54]. Myocytes and adipocytes are the two major cell types involved in the regulation of whole-body metabolism of glucose and lipids, and their dysfunction contributes to the pathogenesis of insulin resistance and type 2 diabetes. Knockdown of SIRT3 by using shRNA to treat C2C12 myoblasts disturbed the differentiation of myocytes including decreases in the fusion index as well as impairment in the induction of expression of myogenin and MyoD [55]. In addition, loss of SIRT3 also leads to defects in mitochondrial biogenesis and respiration, decrease of MnSOD activity and increase of intracellular ROS levels, causing dysfunction of myocytes [55]. Recently, our group explored the role of SIRT3 in the adipogenic differentiation of human adipose tissue-derived mesenchymal stem cells (ad-hMSCs) and found that SIRT3 expression is induced during adipogenic differentiation of ad-hMSCs. SIRT3 deficiency impairs not only the ability of adipogenic differentiation of ad-hMSCs but also the insulin sensitivity of differentiated adipocytes. Mechanistically, the coordination between mitochondrial function and redox homeostasis via the SIRT3-PGC1α/FoxO3A axis is important for differentiation and function of adipocytes [56].

In addition to white adipocytes, SIRT3 regulates in vitro differentiation of brown adipocytes. Brown adipocytes are responsible for consuming excess fuels via thermogenesis. Accumulated evidence has documented that an increase of the amount and activity of brown adipocytes facilitates glucose utilization and combats obesity and diabetes [57,58]. Giralt et al. showed that SIRT3 is required for the activation of the thermogenesis process in brown adipocytes upon the stimulation of β-adrenergic signaling [59]. In addition, the SIRT3 level is increased during differentiation of brown adipocytes and mediates the induction of PGC-1α, and a couple of mitochondria-related genes, and most importantly, uncoupling protein 1 (UCP1) that executes thermogenesis [59]. Considering the contribution of adipocytes in the regulation of whole-body metabolism, the role of SIRT3 in the commitment or differentiation of stem cells into adipocytes may provide a therapeutic potential in treatment of metabolic disorders.

## 3. SIRT4 Regulates Mitochondrial Metabolism and Insulin Sensitivity

In addition to having the deacetylase activity like other sirtuins, SIRT4 mainly acts as an ADP-ribosyl transferase to transfer the ADP-ribose moiety of NAD^+^ to lysine, arginine, glutamate, aspartate, cysteine or serine residues of target proteins. SIRT4 is dominantly expressed in liver, kidney, testis, heart and skeletal muscle. Several studies have shown its roles in the regulation of mitochondrial function, antioxidant defense, insulin secretion and lipid metabolism [16].

### 3.1. SIRT4 in Mitochondrial Function and Redox Homeostasis

It has been reported that SIRT4 modulates ATP production in mitochondria. In vitro cell culture experiments and in vivo mouse models both revealed that SIRT4 deficiency causes a decrease in the intracellular ATP level. In contrast, gain of function by SIRT4 overexpression increases the ATP level in the mammalian cells [60]. Mechanistically, SIRT4 targets adenine nucleotide translocator 2 (ANT2), which is located on the mitochondrial inner membrane and transports ATP and ADP to leave from and enter into mitochondria, respectively. Deacetylation of ANT2 by SIRT4 facilitates the coupling of mitochondrial respiration with oxidative phosphorylation and leads to efficient ATP generation. In addition, impairment of ATP homeostasis by SIRT4 deficiency can trigger the retrograde signaling to activate mitochondrial biogenesis and fatty acids oxidation via the AMPK-PGC1α pathway [61].

However, several studies demonstrated a negative regulatory role of SIRT4 in mitochondrial function and redox homeostasis. It was reported that overexpression of SIRT4 resulted in increase of ROS levels and mitochondrial dysfunction and impaired meiosis and maturation process of mouse oocytes [62]. Moreover, the expression level of SIRT4 is increased in aged oocytes. Deleting SIRT4 in oocytes could partially restore the age-associated defective phenotypes from maternal contribution [62]. Luo et al. highlighted the role of SIRT4 in the pathophysiology of cardiac hypertrophy [63]. Heart-specific SIRT4 transgenic mice displayed serious heart failure, fibrosis and cardiac hypotrophy compared to wild-type mice after treatment with angiotensin II for 4 weeks. Mechanistically, SIRT4 interferes with the interaction between MnSOD and SIRT3, which results in high acetylation and low activity of MnSOD and leads to ROS accumulation. Improvement of redox status by treating mice with a mimetic of SOD can efficiently rescue the defects of SIRT4-mediated cardiac dysfunction [63]. Moreover, SIRT4 regulates the mitochondrial fusion and fission process. SIRT4 physically interacts with the optic atrophy 1 (OPA1) protein to promote the fusion of mitochondria and counteract fission and the mitophagy process. Under the stress conditions, upregulation of SIRT4 causes an imbalance between mitochondrial fusion and fission events and impairs the mitophagy in the removal of the defective mitochondria. SIRT4 sensitizes the stress-induced mitochondrial ROS accumulation and accelerates the vicious cycle of mitochondrial dysfunction under oxidative stress [64].

### 3.2. SIRT4 in Insulin Secretion

Ahuja et al. [65] demonstrated that glucose-stimulated insulin secretion was enhanced in INS1 cells deficient of SIRT4. Mechanistically, SIRT4 adds the ADP-ribosyl group to glutamate dehydrogenase (GDH) and decreases its enzymatic activity. Consequently, inhibition of GDH impairs ATP generation and insulin secretion. It was reported that knockout of SIRT4 and downregulation of SIRT4 by caloric restriction both activate GDH and improve the ability of pancreatic β cells to secrete insulin in response to glucose or amino acids [66]. Consequently, mice displayed better insulin sensitivity and glucose tolerance.

A recent study from Anderson et al. showed that SIRT4 regulates leucine catabolism/oxidation via activation of methylcrotonyl-CoA carboxylase 1 (MCCC1) [67]. Leucine is a branched-chain essential amino acid and plays a crucial role in controlling protein synthesis and regulating cell metabolism in various cell types. In pancreatic β cells, leucine acts as an allosteric activator to stimulate GDH function and insulin secretion [68]. Removing acyl groups by the deacylase activity of SIRT4 allows MCCC1 to contain oxidized leucine residues and to maintain functionally intact. Subsequently, a decrease in the level of leucine leads to reduced GDH activity and impaired insulin secretion. Interestingly, mice lacking SIRT4 resulted in the elevation of basal and glucose-stimulated insulin secretion in pancreatic β cells [67]. A chronic high level of insulin in blood circulation leads to the development of insulin resistance and type 2 diabetes in mice [67].

### 3.3. SIRT4 in Fatty Acid Oxidation

SIRT4 is upregulated in the liver [69] and adipose tissues [70] in rodents after high-fat diet feeding. In addition, patients with non-alcoholic fatty liver disease (NAFLD) exhibited a high level of SIRT4, which was accompanied by upregulation of the expression and activity of lipogenesis-related enzymes/proteins in liver [71]. It has been shown that SIRT4 inhibits the activity of malonyl-CoA decarboxylase (MCD) via deacetylation. MCD is an enzyme involved in fatty acid catabolism to convert malonyl-CoA to acetyl-CoA. Thus, SIRT4 represses fatty acids oxidation but promotes lipogenesis in the liver or adipose tissues. Inhibition of SIRT4 upregulation in obese mice improves fatty acid utilization, exercise performance and protects the animals from liver steatosis [72].

In addition to regulating enzymatic function, SIRT4 also modulates the activities of transcription factors to regulate the expression of genes involved in lipid metabolism. Knockdown of SIRT4 upregulates genes in fatty acid oxidation and enhances the capacity of lipid utilization in the liver, muscle and adipocytes in vivo and in vitro [60,61]. Mechanistically, SIRT4 deficiency induces AMPK activation and SIRT1 upregulation, which coordinates the increase of transcription of PPARα-mediated genes that are involved in the oxidative metabolism of fatty acids such as carnitine palmitoyltransferase 1 (CPT1) and medium-chain acyl-CoA dehydrogenase (MCAD) [73].

## 4. SIRT5 Regulates Mitochondrial Metabolism and Insulin Sensitivity

SIRT5 is one of the mitochondrial sirtuins that regulate mitochondrial respiration and redox homeostasis. SIRT5 also modulates catabolism of different nutrients including glucose, fatty acids and amino acids. Several recent studies demonstrated that SIRT5 plays a regulatory role in the differentiation and function of the BAT, which is responsible for energy expenditure and combat of obesity and diabetes [74,75]. In addition to catalyzing deacetylation, SIRT5 has the enzyme activities of demalonylation, desuccinylation and deglutarylation on a number of mitochondrial proteins by removing malonyl, succinyl and glutaryl groups, respectively, from lysine residues of target proteins [12].

Several tissues with high metabolic activity express a high level of SIRT5 such as brain, skeletal muscle, liver, heart, BAT and kidney, indicating the important role of SIRT5 in metabolic regulation [76]. Unlike SIRT3 and SIRT4, SIRT5 expression is not regulated by caloric restriction and the mice without SIRT5 did not exhibit overt metabolic dysfunction under normal conditions [76]. However, a couple of studies demonstrated the importance of SIRT5-mediated metabolic adaption under stress conditions [14].

### 4.1. SIRT5 in Mitochondrial Function and Redox Homeostasis

Guedouari et al. reported that SIRT5 is required for the regulation of the mitochondrial fusion/fission process. Under the nutrient starvation condition, deletion of SIRT5 increased the level of dynamin-related protein 1 (DRP1) and triggered mitochondrial fragmentation and mitophagy in mouse embryonic fibroblasts [77]. This suggests that SIRT5 protects cells from starvation-induced autophagic degradation [77]. SDHA and PDH have been identified as the targets of SIRT5 in liver by systematic succinylome profiling [78]. Knockdown of SIRT5 results in an increase of succinylation of mitochondrial proteins and inhibited the activities of the proteins leading to subsequent impairment of mitochondrial respiration [78].

Similar to SIRT3, SIRT5 can activate IDH2 to increase NADPH production and ROS scavenging capacity. However, it is mediated by desuccinylation, not by deacetylation [79]. Moreover, SIRT5 directly binds to glucose 6-phosphate dehydrogenase (G6PD) and increases its activity by deglutarylation [79]. G6PD is the rate-limiting enzyme of the pentose phosphate pathway to convert glucose 6-phosphate to ribose 5-phosphate for the biosynthesis of nucleotides and simultaneously produce NADPH for the reduction of oxidized glutathione. Based on the role of SIRT5 in the regulation of IDH2 and G6PD, a lack of SIRT5 decreases intracellular levels of NADPH and GSH, thereby leads to an increase in the intracellular ROS levels and increases the susceptibility of affected cells to oxidative stress [79]. Interestingly, it has been observed that SIRT5 is localized to the cytosol and targets to copper-zinc superoxide dismutase (Cu/ZnSOD) [80]. Overexpression of SIRT5 promotes desuccinylation and activation of Cu/ZnSOD to eliminate cellular ROS [80]. A recent study demonstrated that SIRT5 directly desuccinylates and inhibits peroxisomal acyl-CoA oxidase 1 (ACOX1) [81]. ACOX1 is a rate-limiting enzyme of fatty acid oxidation coupled with H_2_O_2_ production in peroxisomes. Thus, SIRT5 can attenuate oxidative stress induced by peroxisome-derived ROS. Taken together, SIRT5 may regulate redox homeostasis by a multi-directional manner in different cellular compartments including the cytoplasm, mitochondria and peroxisomes.

Pyruvate kinase muscle isozyme 2 (PKM2), which catalyzes the last step of glycolysis to dephosphorylate phosphoenolpyruvate to pyruvate, has been identified as a target of SIRT5 [82,83]. It has been shown that succinylation of PKM2 increases its enzymatic activity. SIRT5 inhibits the activity of PKM2 via desuccinylation at lysine 498. This redirects glucose flux into pentose phosphate pathway instead of glycolysis and leads to an increase of NADPH production. Inhibition of SIRT5 or substitution of succinylated mimetic mutation (K498E) on PKM2 could increase cell death in response to oxidative stress [82]. In contrast, another group observed that SIRT5-mediated desuccinylation of PMK2 impairs its translocation to mitochondria and stabilization of voltage-dependent anion channel 3 (VDAC3), which led to the opening of mPTP and apoptosis under glucose deprivation [83]. The discrepancy from the above two studies may be resulted from different stress conditions (oxidative stress vs. nutrient stress), cell types (lung cancer cells vs. colon cancer cells) or localization of PKM2 (cytosol vs. mitochondria).

### 4.2. SIRT5 in Glycolysis, Fatty Acid Oxidation and Ammonia Metabolism

Using quantitative proteomic analysis, Nishida et al. identified the protein malonylome in the liver cells with or without SIRT5 [84]. Pathway analysis of these demalonylated proteins indicated that glycolysis and gluconeogenesis are the top two SIRT5-regulated pathways. Loss of SIRT5 dramatically abolished glycolytic flux in primary hepatocytes. Genetic manipulation of a malonyl-lysine mimic on lysine 184 residue of glyceraldehyde 3-phosphate dehydrogenase (GAPDH), one of the SIRT5-demalonylated substrates, resulted in inhibition of its enzymatic activity. This finding indicates that SIRT5 may play a role in the regulation of glucose metabolism and in the insulin response [84]. Importantly, it was found that the SIRT5 expression level in adipose tissues was positively correlated with insulin sensitivity in monozygotic twins [85].

Moreover, quantitative protein succinylome studies revealed that SIRT5 desuccinylates a set of mitochondrial proteins that are involved in TCA cycle (IDH2 and GDH), fatty acid metabolism (3-hydroxy-3-methylglutaryl-CoA synthase 2, HMGCS2) and ketogenesis (HMGCS2) in the liver [78,86]. Deletion of SIRT5 results in accumulation of medium- and long-chain fatty acylcarnitines and reduces the production of β-hydroxybutyrate in the liver of mice [86]. Mechanistically, SIRT5 targets and succinylates two critical lysine residues at positions 83 and 310 of HMGCS2, a rate-limiting enzyme in ketogenesis, and increases its activity [86]. In addition to its role in liver, SIRT5 also regulates β-oxidation of fatty acids by protein desuccinylation and thereby protects heart from hypertrophic cardiomyopathy [87]. In humans, the expression of SIRT5 is downregulated in the liver from patients with NAFLD, strengthening the connection between SIRT5 deficiency and defective β-oxidation of fatty acids [71].

Regarding the deacetylase activity, Nakagawa et al. [88] reported that SIRT5 can physically interact with carbamoyl phosphate synthetase 1 (CPS1), which catalyzes the first step in the urea cycle. CPS1 activation by SIRT5-mediated deacetylation promotes the disposal and detoxification of ammonia during fasting. SIRT5 KO mice exhibited a high level of circulating ammonia due to impaired CPS1 activity.

### 4.3. SIRT5 in BAT Function and Adipogenic Differentiation

There are two functionally different types of fat in mammals: brown adipose tissue (BAT) and white adipose tissue (WAT). Both tissues are actively involved in energy homeostasis, but their morphological features, functions and distributions are quite different. BAT expresses exclusively UCP1 to dissipate energy for the generation of heat, known as thermogenesis. WAT has long been recognized as the main site of storage of excess fuels in the form of triglycerides [89,90]. Activation of BAT-induced thermogenesis can provide a natural defense against obesity and diabetes. It has been demonstrated that the level of BAT activity in human adults is negatively correlated to the body mass index [57]. In addition, the amount and function of BAT are associated with a lean and healthy phenotype in animal models [58]. The SIRT5 expression level in BAT is higher than that in WAT [91]. There is a wide spectrum of malonylation and succinylation modifications in mitochondrial proteins of BAT [74], which suggests that BAT plays a crucial role in the regulation of energy expenditure and glucose homeostasis.

Shuai et al. showed that SIRT5 is required for the differentiation of brown adipocytes and browning of white adipocytes in vitro and in vivo [75]. SIRT5 deficiency decreases intracellular α-ketoglutarate level and leads to the accumulation of repressive histone modifications (H3K9me2 and H3K9me3) at the promoter region of *Prdm16*, which is a transcription factor required for the expression of brown adipocyte-specific genes [75]. However, the details of mechanism underlying SIRT5-mediated epigenetic regulation warrant further investigation. In addition, IDH2 produces α-ketoglutarate simultaneously when catalyzing the oxidative decarboxylation of isocitrate. Thus, it is imperative to elucidate whether activation of IDH2 by SIRT5 is involved in the differentiation of brown adipocytes.

Considering the role of SIRT5 in BAT, Wang et al. established a BAT-specific SIRT5 KO mouse model. SIRT5-deficient BAT displayed hyper-succinylation on multiple proteins including GDH, SDHA, and most importantly, the thermogenic protein UCP1. Reduced activities of these proteins and enzymes could lead to metabolic inflexibility, glucose intolerance, impaired cold adaptation and defective mitochondrial function in mice [74].

## 5. Conclusions and Perspectives

Mitochondrial sirtuins, SIRT3-5, respond to nutrient availability and oxidative stress to modulate cellular metabolic homeostasis. Through deacylase or ADP-ribosyl transferase activity, they modulate the functions of multiple proteins and enzymes in mitochondria. Moreover, some evidence also revealed their regulatory roles outside of mitochondria. As we have highlighted in this review, mitochondrial sirtuins are involved in multifaceted cellular metabolism events including glucose metabolism, β-oxidation of fatty acids, amino acid catabolism, mitochondrial respiration, antioxidant defense and insulin secretion (Table 1). Oxidative stress elicited by increased intracellular levels of ROS and free fatty acids can damage mitochondria and decrease the efficiency of oxidative phosphorylation, and results in insufficient supply of ATP required for insulin secretion. Therefore, defects in mitochondrial sirtuins may lead to dysregulated metabolism and, thereby, contribute to the pathogenesis of insulin resistance and type 2 diabetes.

It is noteworthy that the post-translational modifications by mitochondrial sirtuins create a complex network to fine tune metabolic homeostasis. Specificity and overlapping in substrates or modified lysine positions by different sirtuins achieve this complexity since they provide synergistic or opposite effects on protein function or enzyme activities in different metabolic pathways. For instance, SIRT3 and SIRT5 both promote the activities of the same targets (PDH, SDHA and IDH2), but they are affected by different modifications, deacetylation and desuccinylation, respectively. It is worthwhile to investigate the synergistic activation between SIRT3 and SIRT5-mediated regulation on different lysine residues. In addition, SIRT3 and SIRT5 modulate mitochondrial and cytoplasmic ROS levels via MnSOD and Cu/ZnSOD, respectively, to coordinate the maintenance of redox homeostasis. On the other hand, SIRT4 displays an antagonistic effect on the same substrate of SIRT5. Instead of activation by SIRT5-mediated desuccinylation, GDH is inhibited by SIRT4-mediated ADP-ribosylation, causing different outcomes in amino acid catabolism and insulin secretion. Given the interconnected roles of different mitochondrial sirtuins, it is important to consider the independent, synergistic or compensatory impacts on cellular metabolism and the development of diseases such as type 2 diabetes.

As reviewed above, SIRT3 and SIRT5 activate glucose/lipid utilization, insulin sensitivity and reduce oxidative stress and inflammation. Therefore, activation of SIRT3 and/or SIRT5 may be a potential therapeutic strategy for treatment of type 2 diabetes. It has been shown that SIRT1 activation by resveratrol, metformin and other synthetic activators exhibits antidiabetic effects in mouse models and human subjects with obesity or diabetes [92,93]. Further investigation of pharmacological agents targeting SIRT3 and SIRT5 to regulate mitochondrial metabolism may facilitate the development of novel therapies for type 2 diabetes. In addition to upregulation of the protein levels, increase of the NAD^+^ coenzyme to enhance the enzymatic activities of mitochondrial sirtuins is another feasible approach. Alteration of the NAD^+^/NADH ratio in tissue cells is associated with insulin resistance and type 2 diabetes. It has been shown that administration of the precursors of NAD^+^, nicotinamide riboside and nicotinamide mononucleotide [94,95], or inhibitors of NAD^+^ase/CD38 [96,97] to elevate the intracellular NAD^+^ level could improve the insulin sensitivity or other outcome in diabetic mouse models [15,98,99]. However, further systemic studies are necessary to evaluate possible side effects. In summary, the development of advanced approaches targeting individual or different combination of mitochondrial sirtuins may provide useful insights for the design of novel therapies for insulin resistance and type 2 diabetes by regulation of the metabolic health of mitochondria.

## Figures and Tables

**Table 1:** Summary of metabolic functions regulated by mitochondrial sirtuins.

Functions	Proteins	Activities	Sirtuins	Modifications	Outcomes	References
Mitochondrial respiration &oxidative phosphorylation	NDUFA9	Activation	SIRT3	Deacetylation	Increase of Complex I activity	[19]
	SDHA	Activation	SIRT3	Deacetylation	Increase of Complex II activity	[20,47,74]
			SIRT5	Desuccinylation		[78]
	OSCP	Activation	SIRT3	Deacetylation	Increase of Complex V activity	[21]
	ANT2	Activation	SIRT4	Deacetylation	Increase of coupled respiration	[61]
	UCP1	Activation	SIRT5	Desuccinylation	Increase of uncoupled respiration	[74]
Acetyl-CoA availability	PDH	Activation	SIRT3	Deacetylation	Increase of acetyl-CoA	[22]
			SIRT5	Desuccinylation		[78]
	AceCS2	Activation	SIRT3	Deacetylation	Increase of acetyl-CoA	[23,24]
	MCD	Inhibition	SIRT4	Deacetylation	Decrease of acetyl-CoA	[72]
Mitochondrial fusion	OPA1	Activation	SIRT4	Deacetylation	Decrease of mitophagy	[64]
Mitochondrial permeability transition pore (mPTP)	CypD	Inhibition	SIRT3	Deacetylation	Inhibition of mPTP opening	[25]
	PKM2	Inhibition	SIRT5	Desuccinylation	Increase of mPTP opening	[83]
Transcriptional regulator	FoxO3A	Activation	SIRT3	Deacetylation	Increase of adipocyte differentiation	[56]
					Increase of mitophagy	[26]
					Increase of antioxidant enzyme level	[39]
Antioxidant defense	MnSOD	Activation	SIRT3	Deacetylation	Decrease of ROS	[36,37,38]
	Cu/ZnSOD	Activation	SIRT5	Desuccinylation	Decrease of ROS	[80]
	ACOX1	Inhibition	SIRT5	Desuccinylation	Decrease of ROS	[81]
NADPH production	GDH	Activation	SIRT3	Deacetylation	Increase of NADPH	[42]
			SIRT5	Desuccinylation		[74,78]
		Inhibition	SIRT4	ADP-ribosylation	Decrease of NADPH	[66]
	IDH2	Activation	SIRT3	Deacetylation	Increase of NADPH	[40,41]
			SIRT5	Desuccinylation		[78,79]
	G6PD	Activation	SIRT5	Deglutarylation	Increase of NADPH	[79]
	PKM2	Inhibition	SIRT5	Desuccinylation	Increase of NADPH	[82]
	MCCC1	Activation	SIRT4	Deacetylation	Decrease of NADPH	[67,68]
DNA repair	OGG1	Activation	SIRT3	Deacetylation	Increase of DNA repair	[34]
Glycolysis	GAPDH	Activation	SIRT5	Demalonylation	Increase glycolysis	[84]
	PKM2	Inhibition	SIRT5	Desuccinylation	Decrease of glycolysis	[82]
Fatty acid metabolism	LCAD	Activation	SIRT3	Deacetylation	Increase of fatty acid oxidation	[45,46]
	MCAD	Activation	SIRT3	Deacetylation	Increase of fatty acid oxidation	[45,46]
	ACAD9	Activation	SIRT3	Deacetylation	Increase of fatty acid oxidation	[45,46]
	HMGCS2	Activation	SIRT5	Desuccinylation	Increase of ketogenesis	[86]
Urea cycle	CPS1	Activation	SIRT5	Deacetylation	Detoxification of ammonia	[88]

Abbreviations: ACAD9, acyl-CoA dehydrogenase 9; AceCS2, acetyl-CoA synthetase; ACOX1, acyl-CoA oxidase 1; ANT2, adenine nucleotide translocator 2; CPS1, carbamoyl phosphate synthetase 1; Cu/ZnSOD, Cu/Zn-containing superoxide dismutase; CypD, cyclophilin D; FoxO3A, forkhead box O3A; G6PD, glucose 6-phosphate dehydrogenase; GAPDH, glyceraldehyde 3-phosphate dehydrogenase; GDH, glutamate dehydrogenase; HMGCS2, 3-hydroxy-3-methylglutaryl-CoA synthase 2; IDH2, isocitrate dehydrogenase 2; LCAD, long-chain acyl-CoA dehydrogenase; MCAD, medium-chain acyl-CoA dehydrogenase; MCCC1, methylcrotonyl-CoA carboxylase 1; MCD, malonyl-CoA decarboxylase; MnSOD, manganese-containing superoxide dismutase; NDUFA9, NADH:ubiquinone oxidoreductase subunit A9; OGG1, 8-oxoguanine-DNA glycosylase 1; OPA1, optic atrophy 1; OSCP, oligomycin-sensitivity conferring protein; PDH, pyruvate dehydrogenase; PKM2, pyruvate kinase muscle isozyme 2; SDHA, succinate dehydrogenase flavoprotein subunit A; UCP1, uncoupling protein 1.
